# Zeolitic Imidazolate Framework-67-Derived NiCoMn-Layered Double Hydroxides Nanosheets Dispersedly Grown on the Conductive Networks of Single-Walled Carbon Nanotubes for High-Performance Hybrid Supercapacitors

**DOI:** 10.3390/nano15070481

**Published:** 2025-03-23

**Authors:** Yingying Li, Qin Zhou, Yongfu Lian

**Affiliations:** 1 Key Laboratory of Functional Inorganic Material Chemistry, Ministry of Education, School of Chemistry and Materials Science, Heilongjiang University, Harbin 150080, China; 2 School of Food Engineering, Harbin University, Harbin 150086, China

**Keywords:** hybrid supercapacitors, NiCoMn-LDH, ZIF-67, single-walled carbon nanotubes, boron-doped graphene aerogel, nitrogen-doped active carbon cloth

## Abstract

A supercapacitor’s energy storage capability is greatly dependent on electrode materials. Layered double hydroxides (LDHs) were extensively studied as battery-type electrodes because of their 2D structure and quick intercalation/deintercalation of electrolyte ions. However, the energy storage capability for pristine LDHs is limited by their large aggregation tendency and poor electrical conductivity. Herein, a novel NiCoMn-LDH/SWCNTs (single-walled carbon nanotubes) composite electrode material, with ultrathin NiCoMn-LDH nanosheets dispersedly grown among the highly conductive networks of SWCNTs, was prepared via a facile zeolitic imidazolate framework-67 (ZIF-67)-derived in situ etching and deposition procedure. The NiCoMn-LDH/SWCNTs electrode demonstrates a specific capacitance as large as 1704.3 F g^−1^ at 1 A g^−1^, which is ascribed to its exposure of more active sites than NiCoMn-LDH. Moreover, the assembled NiCoMn-LDH/SWCNTs//BGA (boron-doped graphene aerogel) hybrid supercapacitor exhibits a superior capacitance of 167.9 F g^−1^ at 1.0 A g^−1^, an excellent energy density of 45.7 Wh kg^−1^ with a power density of 700 W kg^−1^, and an outstanding cyclic stability with 82.3% incipient capacitance maintained when subjected to 5000 charge and discharge cycles at the current density of 10 A g^−1^, suggesting the significant potential of NiCoMn-LDH/SWCNTs as the electrode material applicable in supercapacitors.

## 1. Introduction

With the fast development of hybrid electric vehicles, wearable electronic devices, and a variety of electronic gadgets [1,2,3], environmentally friendly, efficient, and high-powered energy storage systems have attracted much attention in recent years. As an attractive class of energy-storage devices, supercapacitors (SCs) have the special advantages of fast charging and discharging capability, high power density, and excellent cycling stability [4,5]. However, the lower energy density of SCs than batteries severely hinders their large-scale practical applications in many fields [6]. Thus, extensive efforts have been conducted to enhance SCs’ energy density, either by developing novel electrode materials of remarkable specific capacitance [7], or by assembling asymmetric/hybrid SCs (ASCs/HSCs) to extend the operating voltage window [8]. The efficient promotion of specific capacitance relies on the ingenious design and optimization of electrode materials [8], and ASCs/HSCs, fabricated by electric double-layer capacitive negative and pseudo-capacitive/battery-type positive electrodes, have been demonstrated to be an efficient pathway to broaden the operating voltage window [9,10,11].

In line with the essence of energy storage, capacitive electrode materials could be sorted into the electric double-layer (EDLC), battery-type, and pseudo-capacitor categories [12]. Despite of their poor EDLC specific capacitance, carbonaceous electrode materials have the advantages of an enlarged surface area, outstanding conductivity, and remarkable cycle stability, and, thus, can be widely used in composited electrodes [13]. Conversely, the normal transition metal-based compounds undergo faradaic reactions as battery and pseudo-capacitor electrode materials [14]. Particularly, those transition metal compounds with two-dimensional (2D) nanostructures [15,16,17] have been extensively investigated, because of their short electron and ion transport distance, high specific surface area, and abundant active sites for redox reactions.

As a kind of hydrotalcite-like compound, layered double hydroxides (LDHs) are composed of positive metal host laminates and noncovalent intercalated anions (e.g., ${CO}_{3}^{2-}$, ${NO}_{3}^{-}$, $and\;{SO}_{4}^{2-}$) as well as water molecules [18,19]. The flexible anion exchange and the laminar skeleton of LDHs are particularly good for the intercalation and deintercalation of electrolyte ions, and the hydrophilic nature of LDHs is quite conducive to the diffusion of the electrolyte hydrated ions [20]. Moreover, LDHs have the inherent benefits of a large specific surface area, outstanding capacitive performance, electrochemical activity, and 2D morphology-led exposure of more active sites for redox reactions [21]. In addition, multi-metallic LDHs have the advantages of improved conductivity, multiple valence states, and enhanced electrochemical activity over bi-metallic LDHs [22], which would benefit the improvement of the electrode materials’ capacitance. However, the strong aggregation tendency and limited electric conductivity of LDHs prevent the LDH-based SCs from having high-rated charge/discharge and excellent cycling performances [23,24]. To address these issues, Fu et al. [25] prepared the core–shell structured CuBr_2_@NCC-LDH/CF composite, which demonstrated a specific capacitance of 5460 mF cm^−2^ with prior cycling stability. Pan et al. [26] applied Ac^−^ anions as intercalating ions to adjust the interlayer spacing of NiCo-LDH nanoplatelets. The enlarged interlayer spacing contributes a lot to the stabilization of the LDHs’ α-phase, the efficient electron transport/electrolyte penetration, and the elevation of both rate capability and cycling stability. Aiming at enhancing the inherent low electrical conductivity of LDHs, Zang et al. [27] synthesized 3D PNT@NiCo-LDH hierarchical nanocages by a facile solvothermal method coupled with acid etching treatment, and the as-prepared material showed an improved conductivity, an elongated cycling stability, and an elevated specific capacitance. Yao et al. [28] grew tri-metallic MgCoNi-LDH on graphene and assembled an asymmetric supercapacitor with high stability as well as quite good electrochemical performance. In recent years, the self-supporting interconnected conductive porous networks of carbon nanotubes (CNTs) were applied to anchor various nanostructured LDH materials. Mohammad et al. [29] fabricated a hierarchical NiCoCe-LDH/CNT nanocomposite through a simple hydrothermal procedure, which maintained 85.6% of its initial capacitance after 9000 cycles. The electro-synthesized NiAl-LDH/CNT hierarchical composite exhibited a specific capacitance as large as 1800 F g^−1^ as well as a capacitive retention of 65.8% at 10 A g^−1^ [30]. It ought to be pointed out that almost all of the self-supporting interconnected conductive porous networks were made of multi-walled carbon nanotubes (MWCNTs) in the LDH/CNT composites. Since single-walled carbon nanotubes (SWCNTs) possess fewer defects and larger charge conductivity than MWCNTs, it is expected that SWNTs are more suitable materials to reduce the strong aggregation tendency and to elevate the limited electrical conductivity of LDHs, if they are ingeniously composited with LDHs. Therefore, the elaborate design and fabrication of the hybrid architecture would regulate the morphologies, conductivity, and reaction kinetics of the composited LDH/SWCNTs material, contributing a lot to the screening and optimization of capacitive electrode materials.

Owing to its large surface area, variable metal centers and adjustable pore structure, zeolite imidazole frameworked-67 (ZIF-67) is usually utilized as a self-sacrificing template or metal precursor for the preparation of nanostructured LDHs. Particularly, the unique porous characteristic is inherited by the resultant LDH, shortening the ionic diffusion paths. Bi-metallic MCo-LDHs (M = Mg, Ni or, Co) hierarchical structures were synthesized by adding magnesium (nickel or cobalt) nitrate to ZIF-67 [31]. The NiCo-LDH nanosheet arrays fabricated by Zhao et al. [18] through a facile solvothermal method exhibited a specific capacitance of 2203.6 F g^−1^ at 2 A g^−1^ with outstanding rate performance. Recently, Lu et al. [21] in situ grew tri-metallic NiCoMn-LDH with ZIF-67 as the template on the networks of porous carbon, and the as-prepared electrode material showed a mass capacitance of 2236 F g^−1^ at 1 A g^−1^.

Herein, carboxylated single-walled carbon nanotubes (C-SWCNTs) were incorporated in situ to guide the nucleation of ZIF-67-derived LDHs. Subsequently, a novel NiCoMn-LDH/SWCNTs electrode material was obtained via the chemical etching of H^+^ ions and the co-deposition of metallic ions, in which tri-metallic NiCoMn-LDH nanosheets anchored onto the networks of SWCNTs. The incorporated SWCNTs serve as conductive networks for electron transportation between the surfaces of LDHs and external circuits, thereby overcoming the obstacle of the low intrinsic electrical conductivity of LDH materials. Meanwhile, the NiCoMn-LDHs evolved from ZIF-67 polyhedrons from cross-linked nanosheets under the guidance of SWCNTs, inducing the enlargement of the specific surface area of LDH and the contacting area of the electrode and electrolyte, which are favorable for the exposure of active sites, charge transfer, and the diffusion of electrolyte ions, respectively. The NiCoMn-LDH/SWCNTs composite materials demonstrate remarkable electrochemical properties along with superior rate capability when applied as electrode material for SCs. Moreover, a hybrid SC (NiCoMn-LDH/SWCNTs//BGA HSC) was fabricated with NiCoMn-LDH/SWCNTs as the positive electrode and BGA (boron-doped graphene aerosol) as the negative one. The as-prepared HSC showed a large energy density of 45.7 Wh kg^−1^ at 700 W kg^−1^, and three serially linked HSCs could sustain a red LED on for 135 s, evidencing the actual value of the fabricated HSCs.

## 2. Experimental Section

### 2.1. Materials

All of the following chemicals were of analytical grade and used as received: Mn(NO_3_)_2_*·*6H_2_O, Ni(NO_3_)_2_*·*6H_2_O, Co(NO_3_)_2_*·*6H_2_O, 2-methylimidazole (2-MeIM), and polyvinyl alcohol (PVA, 1799 type) (Shanghai Dibo biotechnology Co., Ltd., Shanghai, China); KOH, H_3_BO_3_, and ethylene glycol (Xilong Scientific Co., Ltd., Shantou, China); polyvinylidene fluoride (PVDF) and activated carbon (Taiyuan LZY Technology Co., Ltd., Taiyuan, China). The following experimental materials were applied as received: carbon cloth (WOS1011) (Willtek Photoelectric Materials Co., Ltd., Suzhou, China); high-purity graphite (Shanghai Xili Carbon Co., Ltd., Shanghai, China); the aqueous dispersion of C-SWCNTs (1.5 mg mL^−1^) (XFNANO Materials Tech Co., Ltd., Jiangsu, China). Deionized water (Shanghai Hitech Instruments CO., Ltd., Shanghai, China) was utilized in all experiments.

### 2.2. Preparation of the ZIF-67/SWCNTs Composite

Typically, 4 mL^−1^ of the aqueous dispersion of C-SWCNTs (1.5 mg mL^−1^) was ultrasonically mixed with 50 mL of methanol (Sonics, VCX 750, Vibra-cell, Newtown, CT, USA) at a temperature of 5 °C. Subsequently, 40 mL of a methanol solution of Co(NO_3_)_2_·6H_2_O (0.1 mol L^−1^) and 25 mL of a methanol solution of 2-MeIM (1.5 mol L^−1^) were successively added, and the resulting mixture was continuously stirred for 30 min. After incubation for 24 h by standing still, the occurred sediment was isolated via vacuum filtration and then subjected to washing with a large amount of ethanol. After drying at a temperature of 80 °C for 12 h, the product named ZIF-67/SWCNTs was achieved. In contrast, ZIF-67 was also prepared without C-SWCNTs in the same procedure.

### 2.3. Synthesis of the NiCoMn-LDH/SWCNTs Composite

The NiCoMn-LDH/SWCNTs nanocomposite was synthesized by the chemical etching of H^+^ ions and the co-deposition of metallic ions. When 400 mg of Ni(NO_3_)_2_·6H_2_O and 400 mg of Mn(NO_3_)_2_·6H_2_O dissolved in 100 mL of anhydrous ethanol, 200 mg of ZIF-67/SWCNTs was added. After ultrasonic treatment for 10 min, the mixture was refluxed at 80 °C for 2 h under the action of magnetic stirring. The solid product was isolated through centrifugation at a speed of 5000 rpm for 5 min and then subjected to rinsing successively with ethanol and deionized water. Finally, the NiCoMn-LDH/SWCNTs composite was achieved after drying at 80 °C for 12 h. For comparison, NiCoMn-LDH was also prepared by the replacement of ZIF-67/SWCNTs with ZIF-67 in the above procedure.

### 2.4. Fabrication of Hybrid Supercapacitor (HSC) Devices

The assembled HSC device is of a classic “sandwich structure”. The positive electrode was made up of NiCoMn-LDH/SWCNTs coated on a piece of nitrogen-doped cloth (NACC) (1 × 1 cm^2^) [32], in which the mass loading amounts of NiCoMn-LDH/SWCNTs were optimized (Figure S1) with a ratio of electro-active material/acetylene black (conductive agent)/PVDF (binder) is 8:1:1, while the negative electrode is the composite of boron-doped graphene aerogel (BGA) [33] and NACC. A piece of PVA/KOH gel (1 × 1 cm^2^) was adopted as the electrolyte and diaphragm. Details in the preparation of the BGA/NACC electrode and the PVA/KOH gel are provided in the Supporting Information. The NiCoMn-LDH/C-SWNTs//BGA flexible HSC devices were packaged with Kapton tape.

### 2.5. Material Characterization

X-ray diffraction (XRD) was conducted on a Bruker (Karlsruhe, Germany) D8-ADVANCE diffractometer with a Cu Kα source under the voltage of 40 kV. X-ray photo electron spectroscopy (XPS) was recorded on a KRATOS (Manchester, England) ULTRA^DLD^ photo electron spectrometer utilizing Al Kα radiation to evaluate the elemental compositions and chemical bonding status of the tested samples. Scanning electron microscopy (SEM) observation was carried out with a Hitachi (Tokyo, Japan) S-4800 scanning electron microscope under the voltage of 5 kV to analyze the morphologies and sizes of the targeted samples. Transmission electron microscope (TEM) and high-resolution transmission electron microscopy (HRTEM) observations were performed on a FEI (Hillsboro, OR, USA) F200X operated at 200 kV. The N_2_ adsorption–desorption isotherms were recorded on a Micromeritics (Norcross, GA, USA) APSP2460 Brunauer–Emmett–Teller (BET) surface analyzer, and the Barrett–Joyner–Halenda (BJH) method was adopted to evaluate pore size distribution. Fourier transform infrared (FT-IR) and Raman scattering spectra were collected on a Spectrum One instrument SHIMADZU (Kyoto, Japan) FTIR-8400S and a Jobin-Yvon (Longjumeau, France) HR800 spectrometer, respectively.

### 2.6. Electrochemical Analyses

Cyclic voltammetry (CV) and galvanostatic charge/discharge (GCD) curves along with electrochemical impedance spectroscopies (EISs) were recorded on a Bio-Logic (Seyssinet-Pariset, France) SP-300n electrochemical workstation system. For a typical three-electrode system, the working electrode is the same as the afore-mentioned positive electrode for the HSC device, and the counter and reference electrodes are a piece of Pt foil (1 × 1 cm^2^) and an Hg/HgO electrode, respectively, with an aqueous solution of KOH (1.0 mol L^−1^) as the electrolyte. Details on the electrochemical calculations are provided in the Supporting Information.

## 3. Results and Discussion

### 3.1. Morphology and Elemental Distribution

**Figure 1:**
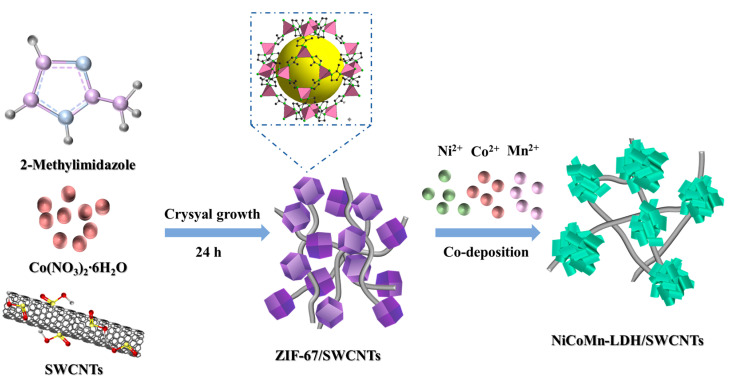
Schematic illustration for the preparation routes of the NiCoMn-LDH/SWCNTs composite derived from zeolitic imidazolate framework.

Figure 1 depicts the preparation of the NiCoMn-LDH/SWCNTs composite. The ZIF-67/SWCNTs composite is initially formed with the growth of crystalline ZIF-67 among the networks of SWCNTs in methanol. Subsequently, the NiCoMn-LDH/SWCNTs composite is obtained via the chemical etching of H^+^ ions on ZIF-67 coupled with the co-precipitation of metallic ions at an elevated temperature.

**Figure 2:**
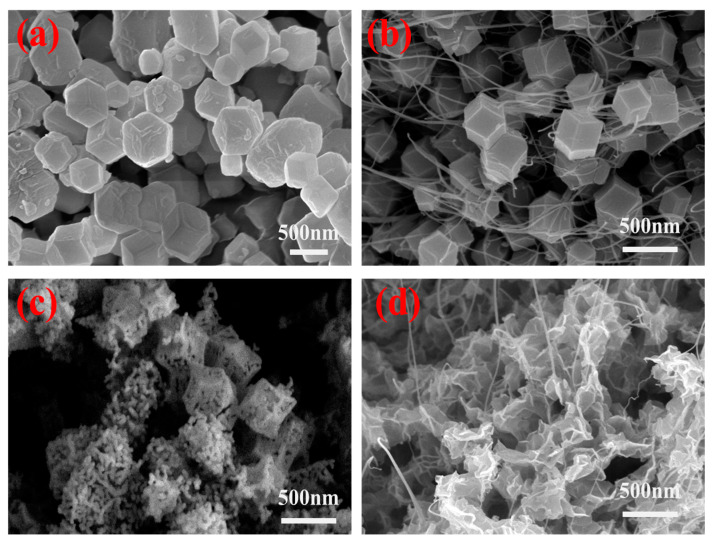
SEM images of (**a**) ZIF-67, (**b**) ZIF-67/SWCNTs, (**c**) NiCoMn-LDH and (**d**) NiCoMn-LDH/SWCNTs.

The morphological evolution of the related materials was investigated by SEM observation. Figure 2a shows clearly that the synthesized ZIF-67 is of a complete rhombic dodecahedron structure with an average size around 560 nm. Whereas, as displayed in Figure 2b, SWCNTs homogeneously intertwine with (some of them penetrate through) ZIF polyhedrons and the average size of ZIF-67 in ZIF-67/SWCNTs is reduced to 330 nm with a size distribution narrower than that of pure ZIF-67 (Figure S2). Such a large decrease in the size and size distribution of ZIF-67 reflects the effect of C-SWCNTs on the growth of crystalline ZIF-67. The negatively charged carboxyl functional groups on SWCNTs could adsorb Co^2+^ ions via electrostatic interactions, and then more nucleation sites are formed on the surface of SWCNTs networks. For a certain amount of Co^2+^ ions and 2-MeIM precursors, the more nucleation sites formed, the smaller the crystal size [34]. On the other hand, the adsorption of Co^2+^ ions on C-SWCNTs yields a more homogeneous distribution in the concentration of Co^2+^ ions, which is responsible for the narrower size distribution of the polyhedrons observed in ZIF-67/SWCNTs than that in pure ZIF-67.

Subsequently, NiCoMn-LDH and NiCoMn-LDH/SWCNTs are prepared with ZIF-67 and ZIF-67/SWCNTs as templates, respectively, via the chemical etching of H^+^ ions and co-precipitation of metallic ions. As shown in Figure 2c, the NiCoMn-LDH derived from a crystalline ZIF-67 template exhibits an irregular polyhedral shape and aggregates to some extent. Even though NiCoMn-LDH inherits the structure of ZIF-67, largely maintaining a rhombic dodecahedron shape, the growth of the NiCoMn-LDH nanosheets is heterogeneous and imperfect, with outer walls obviously shrinking towards the center [21]. In sharp contrast to the crystalline ZIF-67 template, the prepared NiCoMn-LDH has a rough and porous surface. However, as Figure 2d demonstrates, the NiCoMn-LDH/SWCNTs composite derived from the ZIF-67/SWCNTs template exhibits a “blooming flower shape” with a hollow size of several hundred nanometers at the center, which are composed of vertically arranged NiCoMn-LDH nanosheets. Moreover, it is found that SWCNTs intertwine uniformly with, or even penetrate through, the layer-stacked nanosheets of NiCoMn-LDH. Such a significant difference in morphology could be ascribed to the chemical etching effect of H^+^ ions on ZIF-67, in which H^+^ ions are derived either from the hydrolysis of Ni^2+^ and Mn^2+^ ions or from the ionization of the carboxylic acid groups on C-SWCNTs. The released Co^2+^ ions from ZIF-67 and Ni^2+^ and Mn^2+^ ions take part in a co-precipitation reaction with OH^−^ to form NiCoMn-LDH nanosheets among the networks of SWCNTs via an Ostward ripening procedure. The extra H^+^ ions derived from the ionization of carboxylic acid groups on the surface of C-SWCNTs generate a more severe chemical etching effect on ZIF-67 in the NiCoMn-LDH/SWCNTs composite. On the other hand, they also play a role to adjust the concentration of OH^−^, leading to the growth of thinner NiCoMn-LDH nanosheets. The wrinkled ultrathin NiCoMn-LDHs nanosheets are clearly observed to be intertwined uniformly with SWCNTs, which would expose more active sites and be conductive to ion diffusion. Moreover, the highly conductive networks of SWCNTs in NiCoMn-LDH/SWCNTs would significantly enhance the electron migration rate. Thus, excellent capacitive performances are expected for the NiCoMn-LDH/SWCNTs composite electrode material.

**Figure 3:**
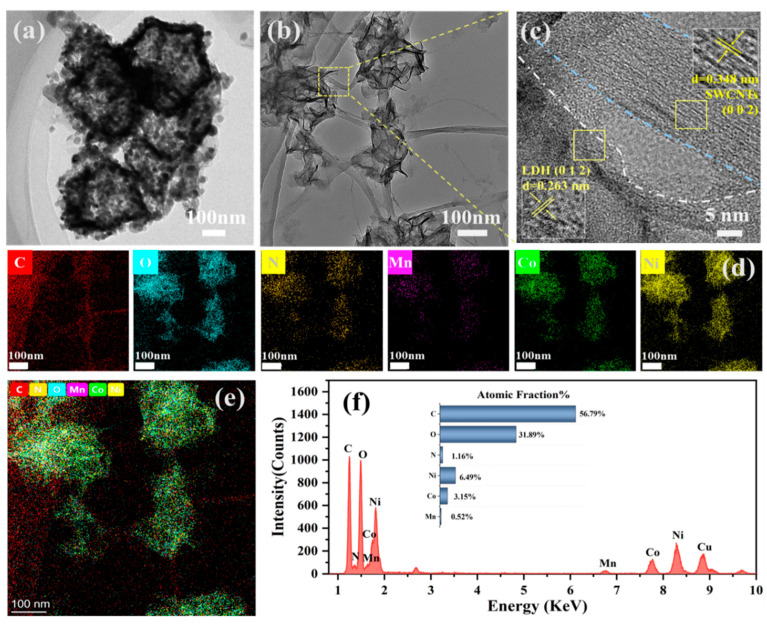
The TEM images of (**a**) NiCoMn-LDH and (**b**) NiCoMn-LDH/SWCNTs along with the (**c**) HR-TEM image, (**d**,**e**) EDS elemental mapping images, and (**f**) EDS analysis of NiCoMn-LDH/SWCNTs.

The micro morphologies and internal structures of the prepared electrode materials were investigated by TEM, HRTEM, and EDS (energy dispersive X-ray spectroscope) characterizations. Figure 3a indicates that the nanosheets of NiCoMn-LDH stacked with each other and a nano-capsid structure is formed. Moreover, a sharp contrast is observed between the shell and core, indicative of the interior cavity of the NiCoMn-LDH nano-capsid. It is estimated that Co ions diffuse out (for the Kirkendall effect) and then co-precipitate with the other two metallic ions to generate NiCoMn-LDH nanosheets, whereas the hollow interior is left behind. The initially formed NiCoMn-LDH would deposit on ZIF-67 [35] to generate a nano-capsid. In line with Zhou et al. [36], the nano-capsid has a big inert cavity and a low packing density, which reduces the effective charge storage and is not conductive to the capacitive performance of NiCoMn-LDH. Whereas well isolated NiCoMn-LDH nanosheets with crimped surfaces and wrinkled edges are observed in the TEM image of NiCoMn-LDH/SWCNTs (Figure 3b), confirming the formation of dispersible and ultrathin-layered NiCoMn-LDH nanosheets, probably owing to the incorporation of SWCNTs. Particularly, it is obvious that those dispersed and thin-layered NiCoMn-LDH nanosheets are intertwined with and closely anchored onto the networks of SWCNTs, which endows the NiCoMn-LDH/SWCNTs composite material with a larger specific surface area and more numerous redox active sites than those of NiCoMn-LDH. SWCNTs play the role of a conducting channel for electron transfer among the NiCoMn-LDH nanosheets, contributing a lot to the enhancement of the conductivity of the NiCoMn-LDH/SWCNTs composite material. Moreover, the lattice fringes observed in Figure 3c correspond to the (012) planes of the NiCoMn-LDH nanosheets and the (002) planes of the SWCNTs, with interplanar distances of 0.263 and 0.348 nm, respectively, confirming the successful preparation of the NiCoMn-LDH/SWCNTs composite electrode material.

EDS is performed to detect the elemental distribution in NiCoMn-LDH/SWCNTs. In line with Figure 3d,e, the N, O, Mn, Co, and Ni elements coexist at the same location of NiCoMn-LDH/SWCNTs, further evidencing the successful preparation of the NiCoMn-LDH/SWCNTs composite. Among them, Mn, Co, and Ni elements are distributed in LDH nanosheets, while O and N elements are derived mainly from the intercalated H_2_O/OH^−^ and ${\mathrm{NO}}_{3}^{-}$ in LDH structure. In contrast, C is mainly derived from C-SWCNTs, which is more uniformly distributed in the composite material, confirming that the NiCoMn-LDH/SWCNTs composite material was successfully prepared and that a higher electronic transport rate as well as an improved electrochemical activity is expected for the composite supercapacitor material. In addition, the atomic ratios of Ni/Co/Mn are estimated to be 1.90:1.00:0.23 and 2.06:1.00:0.17 in NiCoMn-LDH (Figure S3) and NiCoMn-LDH/SWCNTs (Figure 3f), respectively, demonstrating that the proportion of the metallic elements in these products is non-balanced, which might be an effect of the incorporation of C-SWCNTs.

### 3.2. Material Structure

**Figure 4:**
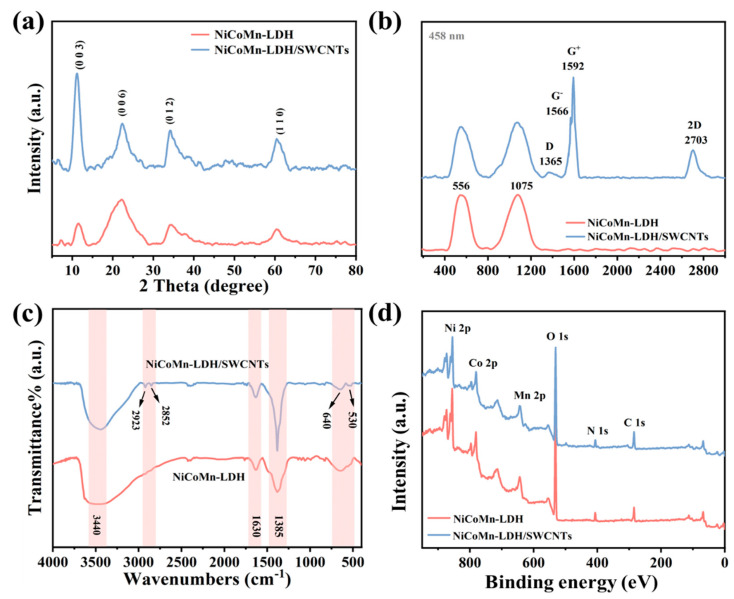
(**a**) XRD patterns, (**b**) Raman (excitation wavelength: 458 nm), (**c**) FT-IR and, (**d**) the survey XPS spectra of NiCoMn-LDH and NiCoMn-LDH/SWCNTs.

XRD was applied to determine the crystalline structure of the prepared electrode materials. Figure 4a shows that the main diffraction peaks are observable at 11.3°, 22.25°, 34.7°, and 60.34°, which could be ascribed to the (003), (006), (012), and (110) crystal planes of the classical LDH structure (JCPDS card no. 33-0429) [37], respectively. Since no extra diffraction peaks are observed, it is concluded that the ZIF-67 template completely converses into LDH material, and that the disappearance of SWCNTs’ diffraction peaks might probably be a result of their low content and good dispersion in the NiCoMn-LDH/SWCNTs composite material. Nonetheless, it should be noted that NiCoMn-LDH and NiCoMn-LDH/SWCNTs exhibit quite different relative intensity for the characteristic (003) and (006) peaks. Ni(OH)_2_·0.75H_2_O shows a typical peaks at 11.3° (JCPDS card no. 38-0715), and Co(OH)_2_ displays a characteristic peak at 19.1° (JCPDS card no. 30-0443) that is coincident partially with the (006) one of LDH. Therefore, the difference in the relative intensity for the characteristic (003) and (006) peaks between NiCoMn-LDH and NiCoMn-LDH/SWCNTs might be owing to their difference in the atomic ratios of Ni/Co. Furthermore, the NiCoMn-LDH/SWCNTs composite displays stronger and narrower diffraction peaks than those of NiCoMn-LDH, implying that C-SWCNTs might promote the growth of the crystalline layered hydrotalcite structure for LDH materials.

To obtain more structural information of the samples, resonant Raman scattering was performed. From Figure 4b, two prominent bands were observed at 556 and 1075 cm^−1^, which could be ascribed to the stretching of M-OH (M = divalent metal ions) bonds [38] and the featured ν1 stretching mode of ${NO}_{3}^{-}$ ions [39], respectively. Moreover, NiCoMn-LDH/SWCNTs present an extra four typical bands, i.e., the D (1365 cm^−1^), G^−^ (1566 cm^−1^), G^+^ (1592 cm^−1^), and 2D (2703 cm^−1^) ones. The split G band (G^−^ and G^+^ bands) is evidence of the existence of SWCNTs [40], whereas the extremely large I_G_/I_D_ ratio and the appearance of the 2D band are both indicative of the SWCNTs’ perfect crystalline structure. From Figure 4c, two bands were detected around 3440 and 1630cm^−1^, which reflect the O-H stretching mode and the bending vibration of the hydrogen-bonded water molecules [11], respectively, originating from the adsorbed water molecules and OH^−^ groups in the layered LDHs. However, the characteristic band detected at 1385 cm^−1^ is due to the asymmetric stretching of ${\mathrm{NO}}_{3}^{-}$ ions in the interlayer of LDH [41]. Thus, it is confirmed that H_2_O molecules and ${\mathrm{NO}}_{3}^{-}$ anions were incorporated into the interlayers of NiCoMn-LDH. Moreover, the characteristic bands observed at 640 and 530 cm^−1^ are generated from the stretching vibration of M-OH/M-O (M= Ni, Co or Mn) in the hydrotalcite-like lattice [21]. Simultaneously, the bands appearing around 2923 and 2852 cm^−1^ originate from the *ν*_C-H_ vibration modes of the phenyl structure [41] in SWCNTs. Since the characteristic Raman scattering and IR absorption features of both NiCoMn-LDH and SWCNTs were detected in NiCoMn-LDH/SWCNTs, it can be concluded that the NiCoMn-LDH/SWCNTs composite was successfully prepared.

### 3.3. Surface Element State

**Figure 5:**
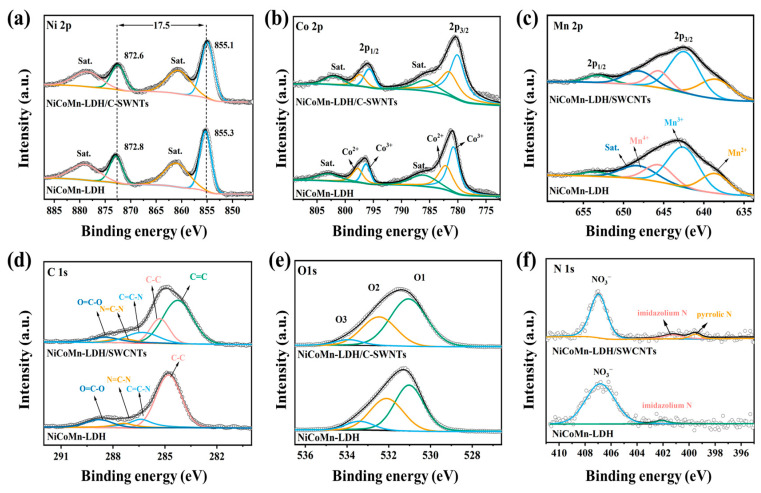
The deconvoluted (**a**) Ni 2p, (**b**) Co 2p, (**c**) Mn 2p, (**d**) C 1s, (**e**) O 1s, and (**f**) N 1s XPS spectra of NiCoMn-LDH and NiCoMn-LDH/SWCNTs.

As indicated in the survey XPS spectra (Figure 4d), the Ni, Co, Mn, O, N, and C elements exist simultaneously in NiCoMn-LDH and NiCoMn-LDH/SWCNTs, which is consistent with the EDS results (Figure 2d). Two prominent peaks along with accompanying satellite peaks could be observed in Figure 5a, which correspond to the 2p_1/2_ and 2p_3/2_ spin–orbit states, respectively. Both NiCoMn-LDH and NiCoMn-LDH/SWCNTs show a spin-energy separation of 17.5 eV, confirming the presence of Ni(OH)_2_ [42]. The deconvoluted Co 2p spectra (Figure 5b) exhibit two spin–orbit bimodal distributions along with two satellites peaks. The peaks around 797 and 782 eV can be attributed to the 2p_3/2_ and 2p_1/2_ spin–orbit states of Co^2+^, and those around 796 and 780 eV to those of Co^3+^ [43], respectively. Moreover, the quite weak satellite peaks around 786 and 802 eV further confirm the +2 and +3 oxidation states of Co element [44]. The deconvoluted Mn 2p XPS spectrum (Figure 5c) demonstrates a weak peak and a broad one at 653.1 and 642.4 eV, which are ascribed to the 2p_1/2_ and 2p_3/2_ spin–orbit states, respectively. Since the magnitude of the splitting decreases with distance from the nucleus due to increased shielding of the nuclear charge, the smaller spin energy separation (10.7 eV) than that of MnO_2_ (11.8 eV) [45] confirms the presence of Mn species with oxidation states less than four. The peak of Mn 2p_3/2_ was fitted to the Mn ions with +2 (638.5 eV), +3 (642.4 eV), and +4 (645.6 eV) oxidation states, respectively, along with the satellite peak of Mn2p_3/2_ (648.1 eV) [46]. The atomic ratios of Mn^2+^/Mn^3+^/Mn^4+^ are evaluated to be 0.44:1.0:0.37 and 0.52:1.0:0.32 in NiCoMn-LDH and NiCoMn-LDH/SWCNTs, respectively, indicating that the predominant Mn species within the LDH are Mn^2+^ and Mn^3+^. It is speculated that the dissolved O_2_ and/or ${\mathrm{NO}}_{3}^{-}$ ions in solution resulted in the oxidation of Co^2+^ and Mn^2+^. No obvious changes in the atom ratios of Co^2+^/Co^3+^ and Mn^2+^/Mn^3+^/Mn^4+^ can be identified in Figure 5b,c, but the corresponding 2p_1/2_ and 2p_3/2_ spin–orbit states in NiCoMn-LDH/SWCNTs slightly shift to a lower bonding energy side relative to those of NiCoMn-LDH, indicating the varied electronic environment between NiCoMn-LDH and NiCoMn-LDH/SWCNTs. As Figure 5d indicates, the deconvoluted C 1s spectrum of NiCoMn-LDH was able to be fitted by four peaks at 284.8, 286.4, 287.4, and 288.8 eV, respectively. Fang et al. [47] ascribed the peak at 284.8 eV to “the abnormal carbon”, since the C 1s spectra of the ubiquitous (adventitious) carbon seem to exhibit an instantaneous presence on all air exposed materials, whereas Lockett et al. [48] ascribed the peak at 284.8 eV to the aliphatic carbons. In the sample of NiCoMn-LDH, apart from “the abnormal carbon” the residual 2-methylimidazole is another carbon source. Because both “the abnormal carbon” and 2-methylimidazole have no sp^2^-type carbon networks, the quite symmetric peak at 284.8 eV is then being fitted to C-C. In contrast, there are some SWCNTs which exist in the sample of NiCoMn-LDH/SWCNTs, and the asymmetrical peak around 284.8 eV is reasonably fitted to the sp^2^-type carbon (284.5 eV) of SWCNs and the sp^3^-type carbon (285.2 eV) of “the abnormal carbon”, 2-methylimidazole, and C-SWCNTs. Meanwhile, the peaks around 286.4 and 287.4 eV were assigned to the aromatic carbon (C=C-N) and the imidazolic carbons (N=C-N) of 2-MeIM [48], respectively, and the peak around 288.6 eV to the O=C-O bond [49] originating from C-SWCNTs, ${CO}_{3}^{2-}$ anions and/or adsorbed CO_2_ in crystalline lattices of LDH. The sp^2^-type carbons of SWCNTs generate conductive networks, while the other oxygen-containing species act as “oxygen bridges” favorable for the electron transportation between NiCoMn-LDHs and SWCNTs, which is a factor in the increase in electrochemical activity of the NiCoMn-LDH/SWCNTs composite. Moreover, in the deconvoluted O 1s XPS spectra (Figure 5e), three main peaks are fitted around 530.5, 531.9, and 533.4 eV, corresponding to the metal–oxygen bonds (Mn/Co/Ni-O), OH^–^ ions in LDH [50], and adsorbed oxygen-containing substances (H_2_O/O_2_/CO_2_) [51], respectively. The atomic content of metal–oxygen bonded O1 in NiCoMn-LDH/SWCNTs increases from 28.8% in NiCoMn-LDH to 31.4%, indicating that SWCNTs promote the nucleation and co-precipitation of metallic ions. For the deconvoluted N 1s spectra (Figure 5f), the prominent peak at 407 eV is ascribed to the N-O bonds in ${NO}_{3}^{-}$ [50], whereas the other ones originate from 2-MeIM residue. For NiCoMn-LDH/SWCNTs, the peaks at 399.6 and 401.4 eV are assigned to imidazolium N [52] and pyrrollic N [53], respectively. It should be noted that the N-O of NiCoMn-LDH/SWCNTs is much narrower than that of NiCoMn-LDH, and that the imidazolium N peak of NiCoMn-LDH is largely blue-shifted relative to that of NiCoMn-LDH/SWCNTs, indicating the various electrostatic interactions between ${NO}_{3}^{-}$ and imidazolium ions in NiCoMn-LDH and NiCoMn-LDH/SWCNTs. Moreover, the presence of the N-O bond also proves the intercalation of ${NO}_{3}^{-}$ in LDH crystalline structure.

### 3.4. Specific Surface Area and Pore Structure

**Figure 6:**
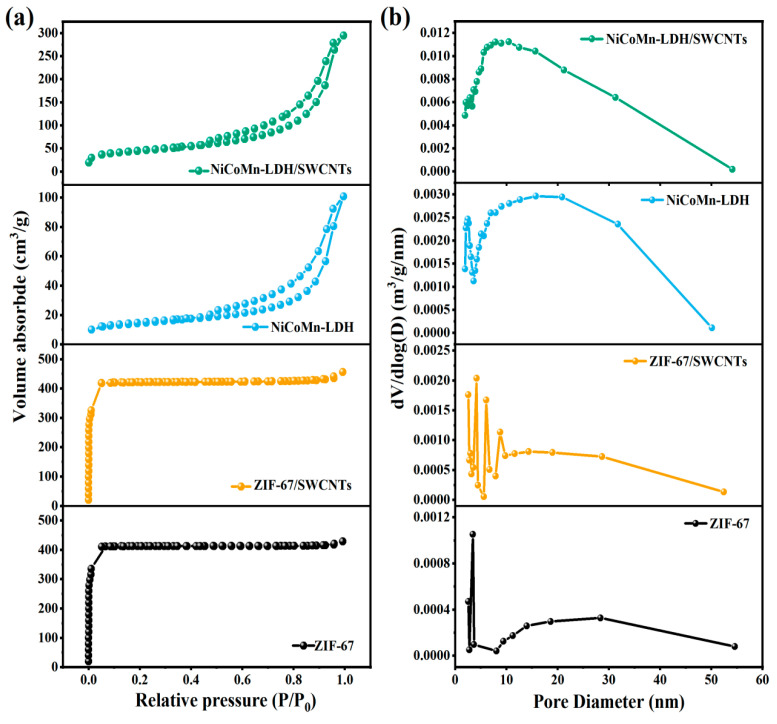
(**a**) N_2_ adsorption–desorption isotherms, and (**b**) BJH pore size distributions of ZIF-67, ZIF-67/SWCNTs, NiCoMn-LDH, and NiCoMn-LDH/SWCNTs, respectively.

It can be seen from Figure 6a that both ZIF-67 and ZIF-67/SWCNTs exhibit characteristic type-I isotherms, which are indicative of their microporous structure. In contrast, both NiCoMn-LDH and NiCoMn-LDH/SWCNTs display the featured type-IV isotherms with H3-type hysteresis loops, which normally originate from the slit-shaped pores out of particle packing, suggesting that the framework of ZIF-67 was reconstituted by chemical etching and the co-precipitation of metal ions and that a slit pore structure was formed with the development of mesopores from micropores. Moreover, the incorporation of SWCNTs with ultrathin NiCoMn-LDH sheets induces a significant increase both in the BET specific surface area and in the pore volume of NiCoMn-LDH/SWCNTs. As displayed in Table S1, the BET specific surface area (157.8 m^2^ g^−1^) and the pore volume (0.43 cm^3^ g^−1^) of NiCoMn-LDH/SWCNTs are much larger than those (51.11 m^2^ g^−1^ and 0.15 cm^3^ g^−1^) of NiCoMn-LDH, implying that more abundant electroactive sites would be exposed for electrochemical reactions. It is of no doubt that such a kind of mesoporous structure would facilitate the diffusion of electrolyte ions and charge transportation, contributing greatly to the electrochemical reaction reactivity and the capacitive storage performance of the NiCoMn-LDH/SWCNTs composite [9]. In line with the BJH pore distribution diagrams displayed in Figure 6b, NiCoMn-LDH/SWCNTs are estimated to have an averaged pore size (14.63 nm) smaller than that of NiCoMn-LDH (17.07 nm), which is appropriate for the accommodation of electrolyte ions, quick diffusion of electrolyte ions, and acceleration of the sluggish kinetics commonly encountered in most energy storage materials involved in a Faradaic redox reaction [27]. Additionally, the pore-size distribution of NiCoMn-LDH/SWCNTs is narrower than that of NiCoMn-LDH, which contributes partly to the enlargement of the specific surface area, efficient charge transport, and electrolyte access. It should be pointed out here that the C-SWCNTs themselves contribute little to the specific surface area of the composite regardless of their specific surface area being as high as 368.18 m^2^ g^−1^ (Figure S4 and Table S1), because the amounts of C-SWCNTs are quite limited and they are well dispersed in the NiCoMn-LDH/SWCNTs composite.

Additionally, in line with a series of CV tests at different scan rates (Figure S5a,b), the number of active sites of NiCoMn-LDH and NiCoMn-LDH/SWCNTs in liquid-phase reactions were reasonably estimated by double-layered capacitances (C_dl_), which have a linear relationship with the electrochemical specific areas (ECSA) [54]. It can be seen from Figure S5c that the C_dl_ value of NiCoMn-LDH/SWCNTs (10.81 mF cm^−2^) is significantly larger than that of NiCoMn-LDH (3.88 mF cm^−2^), confirming the more abundant active sites of NiCoMn-LDH/SWCNTs than those of NiCoMn-LDH. This is consistent with the nitrogen adsorption–desorption results.

### 3.5. Electrochemical Performance of NiCoMn-LDH/SWCNTs

Normally, battery-type electrode materials exhibit obvious oxidation-reduction signals, and the Faraday reactions in alkaline electrolyte for NiCoMn-LDH can be illustrated as follows [19]:

(1) $${Ni(OH)}_{2}{+{OH}^{-}↔NiOOH+H}_{2}O+e^{-}$$

(2) $${Co(OH)}_{2}{+{OH}^{-}↔CoOOH+H}_{2}O+e^{-}$$

(3) $${Mn\left(OH\right)}_{2}{+{OH}^{-}↔MnOOH+H}_{2}O+e^{-}$$

(4) $$MnOOH{+{OH}^{-}↔MnO_{2}+H}_{2}O+e^{-}$$

**Figure 7:**
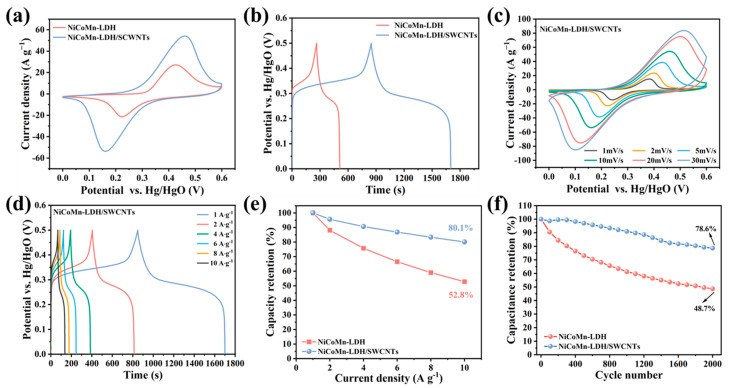
The (**a**) CV curves at 10 mV s^−1^ and (**b**) GCD curves at 1 A g^−1^ of NiCoMn-−LDH and NiCoMn-−LDH/SWCNTs; (**c**) CV curves at various scan rates and (**d**) GCD curves at various current densities of NiCoMn-−LDH/SWCNTs; (**e**) capacitance retention rates at various current densities and (**f**) cycling stability of NiCoMn-−LDH and NiCoMn-−LDH/SWCNTs.

A pair of redox peaks were observed in Figure 7a, which correspond to the Faradaic capacity of M-O/M-OH (M = Ni, Co or Mn). It is obvious that the NiCoMn-LDH/SWCNTs composite exhibits a much greater CV area than that of NiCoMn-LDH, implying the larger specific capacitance and faster reaction kinetics of the composite material. In the GCD plots of NiCoMn-LDH and NiCoMn-LDH/SWCNTs at 1 A g^−1^ (Figure 7b), the observed plateau in the potential range of 0.2–0.4 V evidences the battery-like performance of NiCoMn-LDH and NiCoMn-LDH/SWCNTs [55]. It is obvious that the discharge time of NiCoMn-LDH/SWCNTs is much longer than that of NiCoMn-LDH at the same current density, evidencing the advantageous capacitance of NiCoMn-LDH/SWCNTs over NiCoMn-LDH. For comparison, the CV curves at 10 mV s^−1^, and GCD curves at 1 A g^−1^ of the composites with other ratios of NiCoMn-LDH to SWCNTs were also investigated (Figure S6, Table S2).

All the CV curves shown in Figure 7c exhibit similar forms and symmetric current responses, implying that the composite material has prior rate performance. Simultaneously, the cathodic and anodic responses gradually shift to opposite potentials as the scanning rate increases, which is a result of the current carrier diffusion polarization between electrode material and electrolyte [16], indicating a quick charge–discharge response as well as a remarkable redox reversibility. The mirror-like potential-time responses are observed in Figure 7d, evidencing that the electroactive material has an outstanding columbic efficiency, superior electrochemical reversibility, and high stability, which is understandable by considering that SWCNTs prevent the NiCoMn-LDHs from being eroded by electrolyte [56]. In line with Figure 7d, the specific capacitances of NiCoMn-LDH/SWCNTs are calculated to be 1704.3, 1629.8, 1544.7, 1480.1, 1420.3, and 1365.6 F g^−1^ at 1, 2, 4, 6, 8, and 10 A g^−1^, respectively, indicating the remarkable rate capability of NiCoMn-LDH/SWCNTs. In contrast, those of NiCoMn-LDH are only 496.2, 437.2, 375.8, 330.2, 292.6, and 261.8 F g^−1^ at the corresponding current densities (Figure S7b). Moreover, as Figure 7e indicates, NiCoMn-LDH/SWCNTs maintains 80.1% of its initial capacitance at 10 A∙g−1, which is much superior to that of NiCoMn-LDH (52.8%), further proving the enhanced electrochemical behaviors of NiCoMn-LDH/SWCNTs. Moreover, the CV curve of SWCNTs (Figure S8a) is quasi-rectangular, suggesting the double-layered capacitance behavior of SWCNTs. Additionally, the GCD curve of SWCNTs (Figure S8b) reveals that the specific capacitance at 1 A g^−1^ is only 11.4 F g^−1^. Thus, the contribution from SWCNTs to the capacitance of NiCoMn-LDH/SWCNTs is negligible, and the measured capacitance mainly originates from the NiCoMn-LDH species. As shown in Table S3, the NiCoMn-LDH/SWCNTs electrode exhibits higher specific capacitance than most of the LDH-related materials reported previously, which is a result of its 3D mesoporous structure, ultrathin LDH nanosheets, and the excellent electrical conductivity of SWCNTs networks.

**Figure 8:**
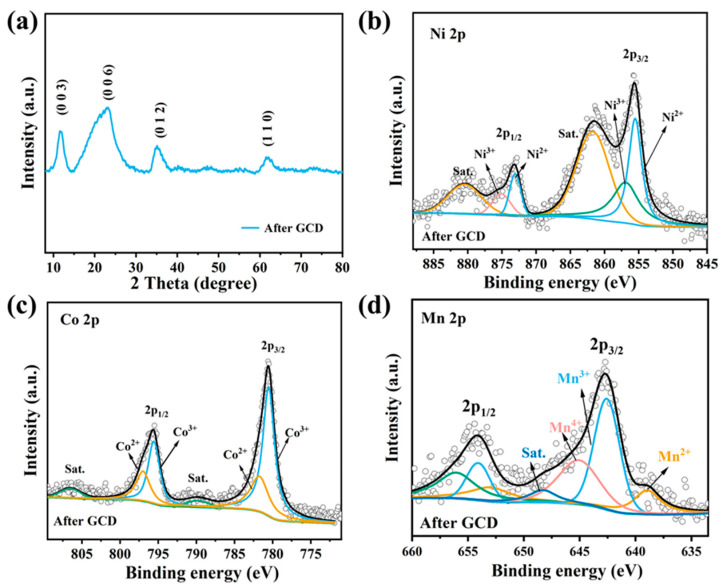
(**a**) XRD pattern and the deconvoluted (**b**) Ni 2p, (**c**) Co 2p, and (**d**) Mn 2p XPS spectra of NiCoMn-LDH/SWNTs after GCD tests.

Cycling stability is also vital for the practical applications of electrode materials, and long-term charge–discharge cyclic tests were applied to estimate the stability of the prepared electrode materials. As Figure 7f shows, NiCoMn-LDH/SWCNTs maintains 78.6% of its initial capacitance after 2000 GCD cycles at 10 A g^−1^, signifying the remarkable cycling performance of NiCoMn-LDH/SWCNTs. In contrast, NiCoMn-LDH maintains only 48.7% of its initial capacitance under the same testing conditions. Therefore, it is concluded that SWCNTs also contribute a lot to the much-extended cycling stability of the NiCoMn-LDH/SWCNTs composite material. Furthermore, XRD and XPS characterizations were performed on the NiCoMn-LDH/SWNTs composite after 2000 GCD cycles to analyze the stability of the composite electrode material. It can be seen from Figure 8a that NiCoMn-LDH/SWNTs maintains the crystalline layered hydrotalcite structure of LDH materials after 2000 GCD cycles, implying the excellent stability of the NiCoMn-LDH/SWCNTs electrode material. Nonetheless, it is noted that the diffraction peaks of NiCoMn-LDH shift a little to higher angles relative to those before GCD tests (Figure 4a), which could be ascribed to the lattice contraction after GCD cycles. Moreover, the peak area and half-peak width of the (006) diffraction peak are larger than those before GCD cycles, which might be due to the lattice distortion-led orientation growth and internal stress of crystal planes, respectively. Shown in Figure 8b–d are the deconvoluted Ni 2p, Co 2p, and Mn 2p XPS spectra of NiCoMn-LDH/SWNTs after GCD tests. In the Ni 2p XPS spectra, the peaks at 875.1 and 857.5 eV correspond to the 2p_1/2_ and 2p_3/2_ spin–orbit split peaks of Ni (III), confirming the transition from Ni (II) to Ni (III). For the Co 2p XPS spectra, the atomic ratio of Co (III)/[Co (III) + Co (II)] increases 8.12% after GCD tests relative to that before GCD tests, evidencing the transition from Co (II) to Co (III). Moreover, no spin–orbit split peaks of Co (IV) were detected, indicating that the transition from Co (III) to Co (IV) does not involve the energy storage mechanism. As for the Mn 2p XPS spectra, the atomic ratios of Mn^2+^/Mn^3+^/Mn^4+^ are evaluated to be 0.30:1.0:0.74 after GCD. In comparison with that before GCD tests (0.52:1.0:0.32), it is obvious that the total content of Mn (IV) increases from 18% to 36%, indicating that the transition from Mn (III) to Mn (IV) is involved in the energy storage mechanism. Furthermore, the larger spin energy separation (11.4 eV) after GCD tests strongly supports that the main oxidation states of Mn are +3 and +4, which is largely aligned with the previously reported results [57]. On one hand, these gradually generated active high-valence NiOOH, CoOOH, MnOOH, and MnO_2_ active phases might be responsible for the lattice contraction of NiCoMn-LDH after GCD tests. On the other hand, electrochemical oxidation/reduction only brings about the redistribution in the oxidation states of Ni, Co, and Mn ions to some extent for the NiCoMn-LDH/SWNTs composite material, further indicating the excellent stability of the composite electrode material. In the crystalline structure of NiCoMn-LDH, the cationic hydroxide layers are made of saturated and symmetric octahedral M(OH)_6_ (M = Ni, Co or Mn) units with an edge-sharing configuration, which prevents LDH materials from structure reconstruction, ion leaching, OH^−^ attack, or dehydrogenation [58].

**Figure 9:**
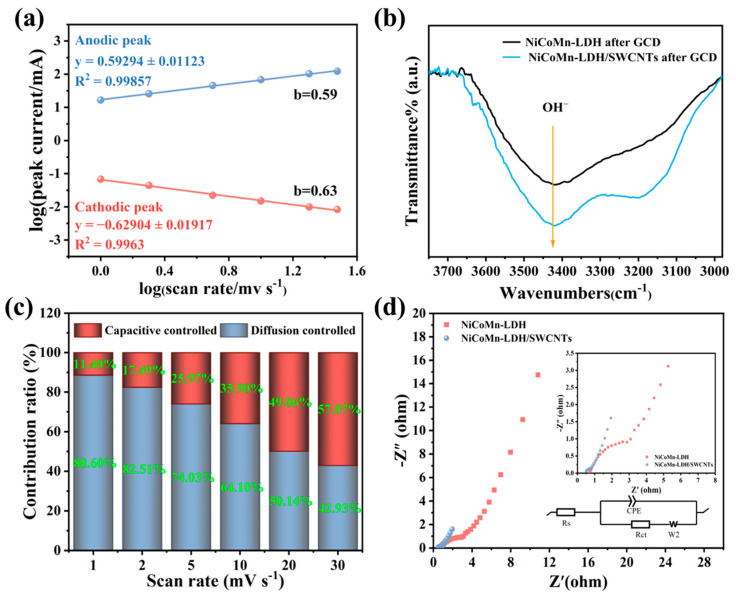
(**a**) Linear fitting plots of log (i) versus log (v) of NiCoMn-LDH/SWCNTs; (**b**) FTIR spectra of NiCoMn-LDH and NiCoMn-LDH/SWCNTs after GCD tests; (**c**) scanning rates dependent diffusion-controlled and capacitive contributions of NiCoMn-LDH/SWCNTs, and (**d**) Nyquist plots of NiCoMn-LDH and the NiCoMn-LDH/SWCNTs (insets are enlarged EIS spectra and the equivalent circuit, respectively).

For a group of systemic CV tests, peak current (i) correlates with scan rate (v) by the following equations [9]:

(5) $$i={\alpha v}^{b}$$

(6) $$\log\left(i\right)=\log\left(\alpha \right)+b\log\left(v\right)$$

where parameters α and *b* could be determined by the linear plot of log⁡(i) vs. log⁡(v), and the *b* value reflects that the charge storage is a battery-like (*b ≈* 0.5) or pseudocapacitive (*b ≈* 1.0) behavior [18,59]. As Figure 9a depicts, the anodic *b* is 0.59, while the cathodic one is 0.63, implying that the charge storage is a similar behavior to a kind of battery-type material, i.e., the charge storage is mainly based on the intercalation/deintercalation of electrolytic ions (OH^−^) in the NiCoMn-LDH/SWCNTs composite material. In line with Guan et al. [58], Fourier transform infrared (FTIR) could be applied to estimate the OH^−^ adsorption capability on electroactive materials. It can be seen from Figure 9b that both NiCoMn-LDH and NiCoMn-LDH/SWCNTs electrode materials present a broad band around 3440 cm^−1^, which reflects the O-H stretching mode of OH^−^ groups in the interlayers of NiCoMn-LDH. Because the band intensity of NiCoMn-LDH/SWCNTs is stronger than that of NiCoMn-LDH after GCD tests, it is concluded that NiCoMn-LDH/SWCNTs have a more efficient OH^−^ adsorption capability than NiCoMn-LDH [60]. The insertion/adsorption of OH^−^ ions in the interlayers of the LDH crystalline structure is beneficial for the gradual generation of the active high-valence NiOOH, CoOOH, MnOOH, and MnO_2_ active phases via electrochemical oxidation other than the OH^−^ attack-led dehydrogenation process [58]. On the other hand, the incorporation of C-WCNTs not only induces more exposure of active sites to OH^−^ ions for NiCoMn-LDH/SWCNTs but also contributes to the insertion/adsorption of OH^−^ ions via the hydrogen bonds between OH^−^ ions and the carboxyl group on the surface of C-WCNTs.

On the other hand, the capacitive ($k_{1}v$) and diffusion-controlled (${k_{2}v}^{1/2}$) contributions to current response could also be distinguished in line with the below equation [28]:

(7) $$i(v)=k_{1}v+{k_{2}v}^{1/2}$$

where the constants $k_{1}$ and $k_{2}$ could be determined by linear fitting i(v)/$v^{1/2}$ vs. $v^{1/2}$. In line with the fitted CV curves at various scan rates (Figure S9), the contributions from diffusion-controlled and capacitor-like processes are distinguished. It can be seen from Figure 9c that the diffusion-controlled process contributes 88.60% at 1 mV s^−1^, and the contribution rate still maintains 42.93% even at 30 mV s^−1^, evidencing the diffusion-controlled charge storage kinetics.

EIS was determined to investigate charge transport kinetics. As Figure 9d displays, the equivalent series resistance (*R*_s_) [61] of NiCoMn-LDH/SWCNTs is as low as 0.51 Ω and the extremely small semicircle of NiCoMn-LDH/SWCNTs reveals its significantly low charge-transfer resistance (*R*_ct_) [25], confirming the superior ion mobility and excellent charge transfer of NiCoMn-LDH/SWCNTs, respectively. In comparison with NiCoMn-LDH, NiCoMn-LDH/SWCNTs display a lower value of *R*_s_, a smaller semicircle diameter and a greater vertical line, indicating that the NiCoMn-LDH/SWCNTs composite has the advantages of a much smaller bulk resistance, a swifter charge transport, and a more efficient charge diffusion [50] over NiCoMn-LDH, which should be a result of the outstanding electrical conductivity of SWCNTs.

Based on the above electrochemical tests, NiCoMn-LDH/SWCNTs exhibited larger specific capacitance, higher rate performance, lower resistance and longer cycle stability than NiCoMn-LDH. These results can be understood from the following viewpoints. Firstly, the NiCoMn-LDHs grown on the networks of SWCNTs are porous, wrinkled, and ultrathin nanosheets, endowing the composite material with abundant accessible surface areas and numerous redox active sites. Secondly, conductive SWCNTs intimately contact with electroactive NiCoMn-LDHs sheets, not only preventing the NiCoMn-LDH nanosheets from severe aggregation but also promoting the charge transfer and redox reaction. Finally, the 3D interconnected networks of NiCoMn-LDH/SWCNTs could sustain the variation in volume and then contribute greatly to the charge/discharge cycling stability [50].

### 3.6. Supercapacitor Testing

**Figure 10:**
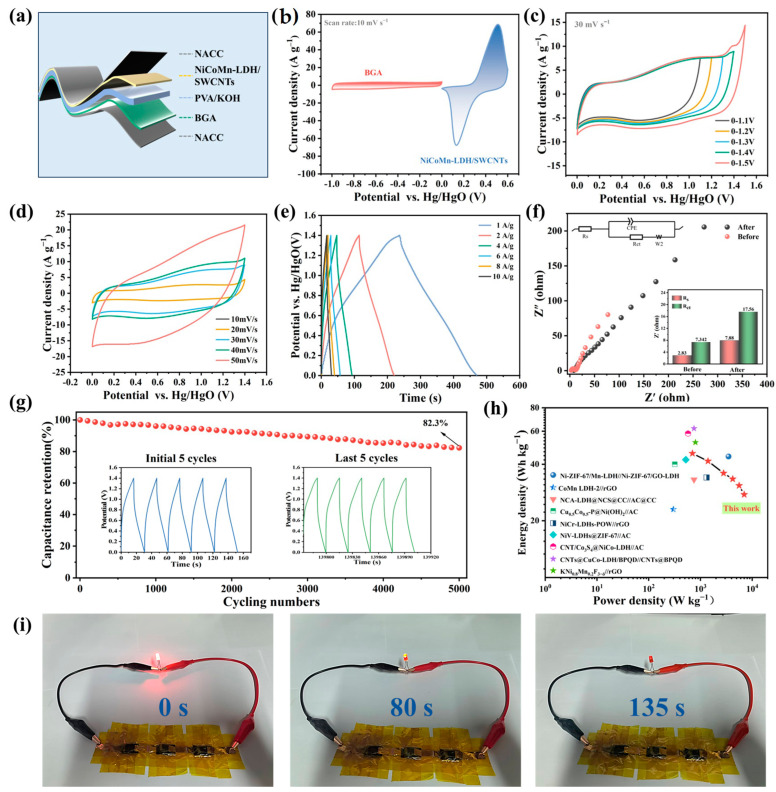
(**a**) The sandwich structure of a fabricated HSC device; (**b**) CV curves of BGA and NiCoMn−LDH/SWCNTs; the HSC device’s (**c**) CV curves at various potential windows; (**d**) CV curves at various scan rates; (**e**) GCD curves at various current densities; (**f**) the Nyquist diagram before and after 5000 cycles; (**g**) the cyclic stability at a current density of 10 A g^−1^ for 5000 GCD cycles (insets are the initial five and the last five cycles, respectively); and (**h**) Ragone plots in comparison with other SCs; (**i**) the photographic images of three serially connected HSC devices that can light up a red LED.

Aiming at evaluating the utilization of the NiCoMn-LDH/SWCNTs composite in practical energy storage, an all-solid-state flexible HSC device was assembled. Shown in Figure 10a is the classical sandwich structure of the device (denoted as NiCoMn-LDH/SWCNTs//BGA). It can be seen from Figure 10b that there is no polarization for either BGA or for NiCoMn-LDH/SWCNTs in their corresponding potential ranges. Moreover, it can also be seen from Figure 10c that no polarization is observable, even at the potential of 1.4 V, even though an apparent polarization occurs at 1.5 V. Therefore, the assembled NiCoMn-LDH/SWCNTs//BGA HSC devices are stable in the working potential range from 0 to 1.4 V. As Figure 10d indicates, all of the CV curves in the potential window of 1.4 V display similar shapes without apparent distortion, implying the good interface dynamics, a superior rate capability, and excellent reversibility of the energy storage device [21]. Meanwhile, all CV curves at different scan rates exhibit a characteristic combination of capacitive and battery-type behaviors. The nearly symmetrical GCD curves observed in Figure 10e confirm the steady charging–discharging conversion of the NiCoMn-LDH/SWCNTs//BGA HSC devices [42], whereas the nonlinearity of these curves is a result of Faradaic electrochemical reactions. The specific capacitances of the NiCoMn-LDH/SWCNTs//BGA device are calculated to be 167.88, 152.74, 131.66, 122.65, 113.14, and 101.35 F g^−1^ at 1, 2, 4, 6, 8, and 10 A g^−1^, respectively, confirming the remarkable capacitance retention and rate capability of the fabricated device. Based on the Nyquist plots shown in Figure 10f, it is estimated that the *R*_s_ and *R*_ct_ of the HSC device are only 2.83 and 7.34 Ω cm^−2^, respectively, indicating a good conductivity and an efficient charge transfer.

It can be seen from Figure 10g that the HSC device retains 82.3% of its capacitance after 5000 cycles at 10 A g^−1^, confirming its excellent redox stability. Shown in the inset of Figure 10g are the initial five and the last five GCD curves. Since no evident differences are observed for these GCD curves, the NiCoMn-LDH/SWCNTs//BGA HSC device exhibits an excellent cycling life. The EIS of the HSC device was also measured to further check the device’s stability. As Figure 10f displays, *R*_s_ and *R*_ct_ of the HSC device increase justly from 2.83 to 7.88 Ω cm^−2^ and from 7.34 to 17.56 Ω cm^−2^, respectively, and the linear components are slight deviated from each other before and after 5000 GCD cycles, further supporting the long-term cyclic stability and superior electrochemical reversibility of the assembled NiCoMn-LDH/SWCNTs//BGA HSC device.

The overall behaviors of the HSC system were estimated by the correlation between energy density and power density. In line with the calculated Ragone plot displayed in Figure 10h, the energy density of the NiCoMn-LDH/SWCNTs//BGA HSC device reaches 45.7 Wh kg^−1^ at a power density of 700 W kg^−1^, and it still maintains 27.6 Wh kg^−1^ at a power density of 7000 W kg^−1^. The fabricated NiCoMn-LDH/SWCNTs//BGA HSC matches or even exceeds many LDH-based SCs reported previously, e.g., NiV-LDHs@ZIF−67//AC [62], Cu_0.5_Co_0.5_-P@Ni(OH)_2_//AC [63], CoMn LDH-2//rGO [64], NCA-LDH@NCS@CC//AC@CC [65], and NiCr-LDHs-POW//rGO [66] (Figure 10h and Table S4). To evidence the practicability of the fabricated NiCoMn-LDH/SWCNTs//BGA flexible HSC device, three identical HSC devices are series-wound, which can maintain a red LED on for 135 s (Figure 10i). Thus, it is concluded that the fabricated NiCoMn-LDH/SWCNTs//BGA flexible HSC devices have strong practicability.

## 4. Conclusions

The NiCoMn-LDH/SWCNTs composite electrode material was prepared via a facile ZIF-67-derived in situ synthesis procedure, in which ultrathin NiCoMn-LDH nanosheets grew closely on the highly conductive and interpenetrating networks of 1D SWCNTs. In comparison to NiCoMn-LDH nanosheets, the NiCoMn-LDH/SWCNTs composite displays significantly improved specific capacitance, cycle stability, and rate performance. The mass capacitance of the composite material reaches 1704.3 F g^−1^ at 1 A g^−1^, with 78.6% of its initial capacity maintained after 2000 GCD cycles at 10 A g^−1^. Furthermore, the assembled NiCoMn-LDH/SWCNTs//BGA flexible HSC devices demonstrate quite good capacitance performance. The capacitance of the fabricated HSC device reaches as high as 167.9 F g^−1^ at 1.0 A g^−1^, and 82.3% of its initial capacity is maintained after 5000 GCD cycles at 10 A g^−1^. The energy density of the assembled HSC device can reach 45.7 Wh kg^−1^ at the power density of 700 W kg^−1^. Three serially connected flexible HSC devices can successfully keep a red LED on for 135 s. Thus, the ingeniously designed NiCoMn-LDH/SWCNTs composite is practically applicable as an appropriate electrode material for the fabrication of advanced HSC devices.

## Data Availability

Data are contained within the article and Supplementary Materials.

## Supplementary Materials

The following supporting information can be downloaded at: https://www.mdpi.com/article/10.3390/nano15070481/s1, Figure S1. The specific capacitance of NiCoMn-LDH/SWCNTs at various mass loading amounts; Figure S2. The size distributions of (a) ZIF-67 and (b) ZIF-67/SWCNTs; Figure S3. EDS spectrum and atomic fraction of NiCoMn-LDH; Figure S4. (a) N_2_ adsorption-desorption isotherm and (b) BJH pore size distribution SWCNTs; Figure S5. Electrochemical CV curves at various scan rates for (a) NiCoMn-LDH and (b) NiCoMn-LDH/SWCNTs along with (c) the calculated electrochemical double-layer capacitance (Cdl) of NiCoMn-LDH and NiCoMn-LDH/SWCNTs; Figure S6. The (a) CV curves at a scan rate of 10 mV s^−1^ and (b) GCD curves at a current density of 1 A g^−1^ for NiCoMn-LDH and NiCoMn-LDH/SWCNTsx (x = 2, 4, 8), respectively; Figure S7. (a) CV curves at 1−30 mV s^−1^ and (b) GCD curves at 1−10 A g^−1^ of NiCoMn-LDH; Figure S8. (a) CV curves at 5−30 mV s^−1^ and (b) GCD curves at 1−10 A g^−1^ of SWCNTs; Figure S9. CV curves showing the diffusion contribution for the NiCoMn-LDH/SWCNTs electrode at various scan rates; Table S1. The BET surface area, average pore size and pore volume of the tested samples; Table S2. Specific capacitances of NiCoMn-LDH and NiCoMn-LDH/SWCNTsx (x = 2, 4, 8) at various current densities; Table S3. The capacitive performance of the LDH based electrode materials; Table S4. The capacitive performance of the HSC devices reported recently.
