# Supplementary material for: Zeolitic Imidazolate Framework-67-Derived NiCoMn-Layered Double Hydroxides Nanosheets Dispersedly Grown on the Conductive Networks of Single-Walled Carbon Nanotubes for High-Performance Hybrid Supercapacitors
Source: Nanomaterials (Basel). 2025 Mar 23;15(7):481. doi: 10.3390/nano15070481 (PMC11990305; doi:10.3390/nano15070481)
Supplement: Supplementary file 1 [file nanomaterials-15-00481-s001.zip › nanomaterials-3498690-supplementary.pdf]

Supporting information

Zeolitic imidazolate framework-67 derived NiCoMn-LDH nanosheets dispersedly grown on the conductive networks of single-walled carbon nanotubes for high-performance hybrid supercapacitors

Yingying Li[a,b], Qin Zhou[a,*], Yongfu Lian[a,*]

[a] Key Laboratory of Functional Inorganic Material Chemistry, Ministry of Education, School of Chemistry and Materials Science, Heilongjiang University, Harbin 150080, China.

[b] School of Food Engineering, Harbin University, Harbin 150086, China

*corresponding authors: zhouqin@hlju.edu.cn, chyflian@hlju.deu.cn

1. Synthesis of BGA/NACC electrode material

BGA/NACC was prepared according to the method reported previously [1]. In brief, H_3BO_3 (230 mg) was added into 25 mL aqueous dispersion of graphene oxide (GO) (2 mg/mL). After stirring for 30 min at room temperature, the mixture and a piece of NACC (1*1 cm) was transferred to a Teflon-lined stainless steel autoclave and hydrothermally treated at 180 °C for 12 h. When the autoclave cooled down to ambient temperature, the product was collected and soaked in an appropriate amount of aqueous solution of ethanol (20%) for 12 h. Finally, the BGA/NACC was obtained after freeze-drying treatment of the product for 48 h.

2. Synthesis of PVA/KOH hydrogel electrolyte

Typically, 6 g of PVA was added into 50 mL of deionized water and magnetically stirred for 30 min, and then continuously stirred at a temperature of 95 °C in oil bath for 3 h. After a uniform and transparent solution formed, 10 ml of 1 M KOH solution

was slowly added dropwise at the temperature 60 °C. When the solution mixture was cooled to room temperature, a transparent PVA/KOH gel was obtained. Finally, the PVA/KOH gel was cured at a temperature of -18 °C in a refrigerator for 24 h to get the PVA/KOH hydrogel electrolyte.

3. Electrochemical calculations.

The specific capacitance of the electrode materials can be calculated from the GCD curves according to the formula.

$$C = \frac{I\Delta t}{m\Delta V} \tag{1}$$

Where I is the discharge current (A), Δt is the discharge time (s), ΔV is the potential window (V) and m is the mass of the active material in the electrode (g). For the two-electrode configuration, the mass loading ratio of anode and cathode electrodes is based on the charge balance theory according to:

$$\frac{m_+}{m_-} = \frac{C_- \times V_-}{C_+ \times V_+} \tag{2}$$

Where, m is the mass of the electrode material (g), C is the specific capacitance of the electrode material (F g^{-1}), and V is the potential window of the electrode.

The energy density and power density of the flexible HSC device were calculated according to the following equations (4) and (5), respectively [2].

$$\mathrm{E}=\frac{1}{2}\cdot\frac{\mathrm{C}\times\Delta\mathrm{V}^2}{3.6} \tag{3}$$

$$P = \frac{3600 \times E}{\Delta t} \tag{4}$$

C is the gravimetric specific capacitance (F g^{-1}), ΔV is the operating voltage (V), Δt is discharge time of the device(s).

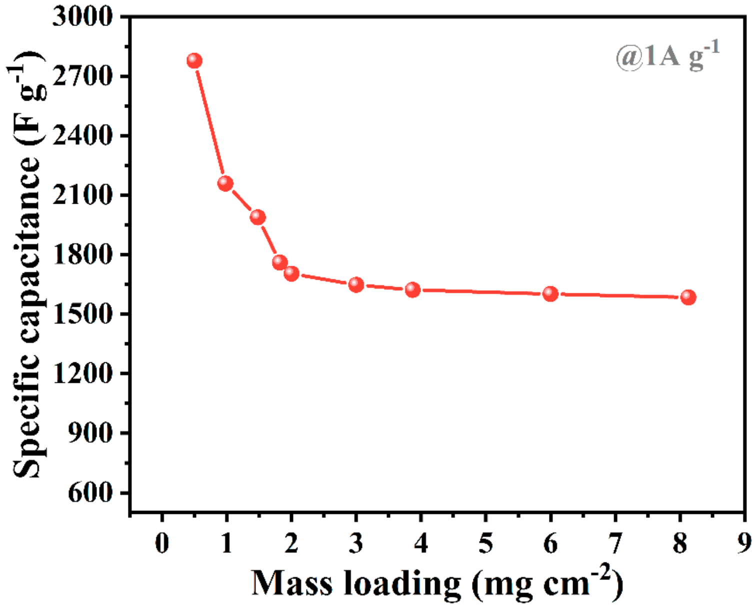

Figure S1. The specific capacitance of NiCoMn-LDH/SWCNTs at various mass loading amounts.

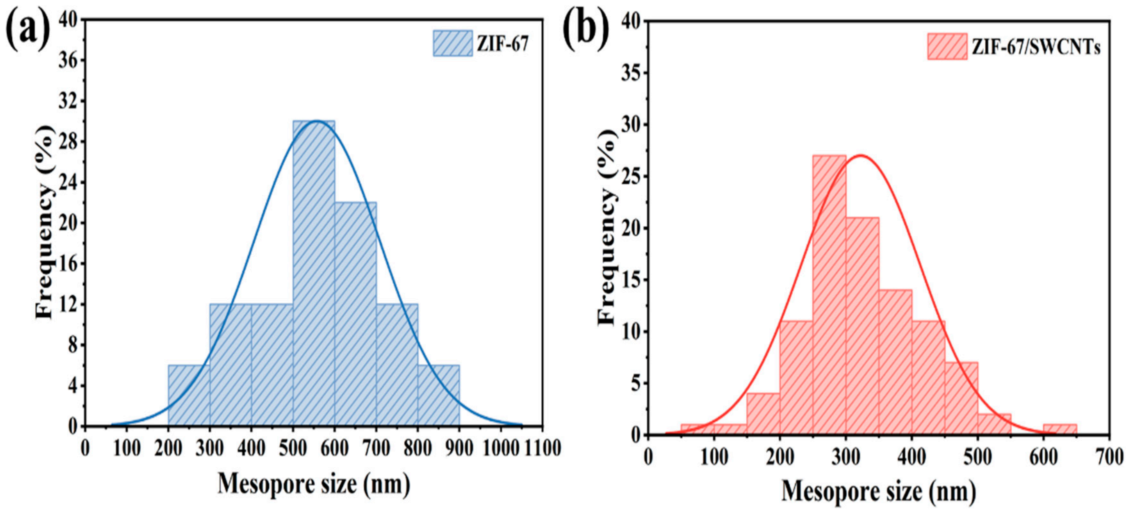

Figure S2. The size distributions of (a) ZIF-67 and (b)ZIF-67/SWCNTs.

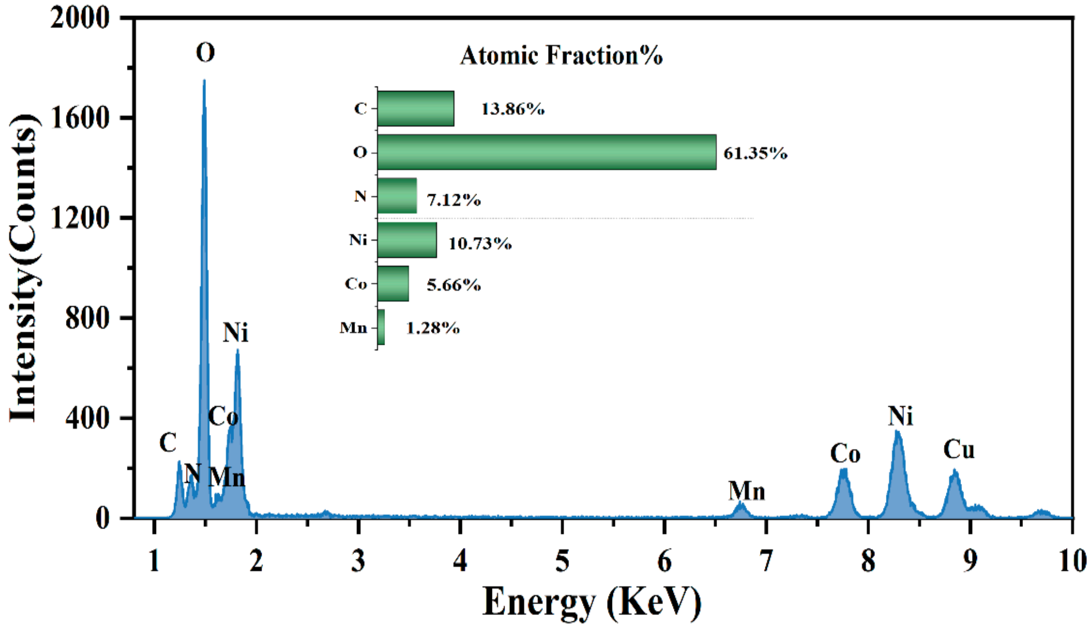

Figure S3. EDS spectrum and atomic fraction of NiCoMn-LDH.

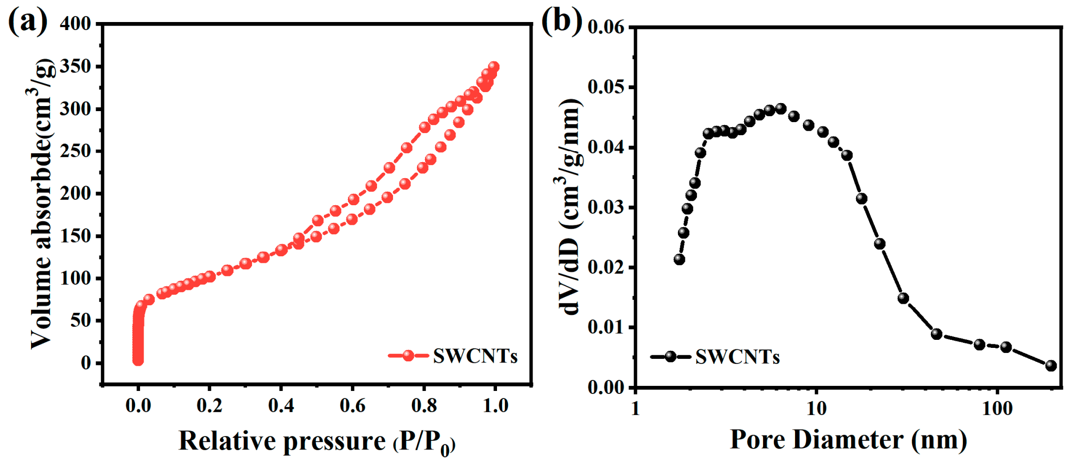

Figure S4. (a) N_2 adsorption-desorption isotherm and (b) BJH pore size distribution SWCNTs.

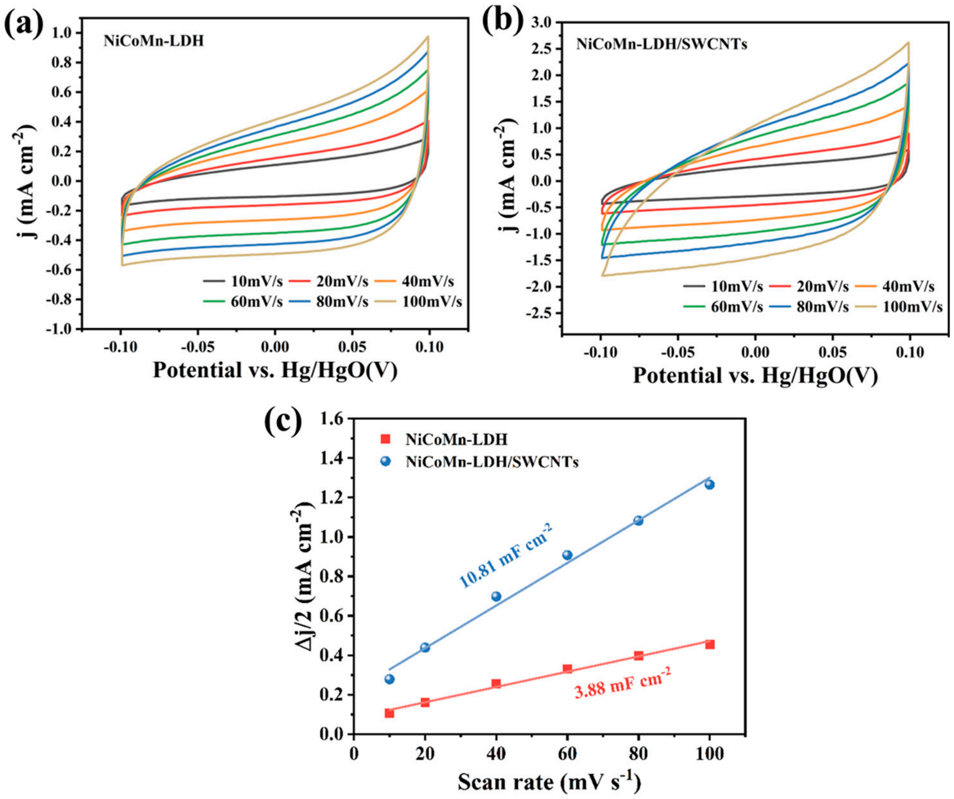

Figure S5. Electrochemical CV curves at various scan rates for (a) NiCoMn-LDH and (b) NiCoMn-LDH/SWCNTs along with (c) the calculated electrochemical double-layer capacitance (Cdl) of NiCoMn-LDH and NiCoMn-LDH/SWCNTs.

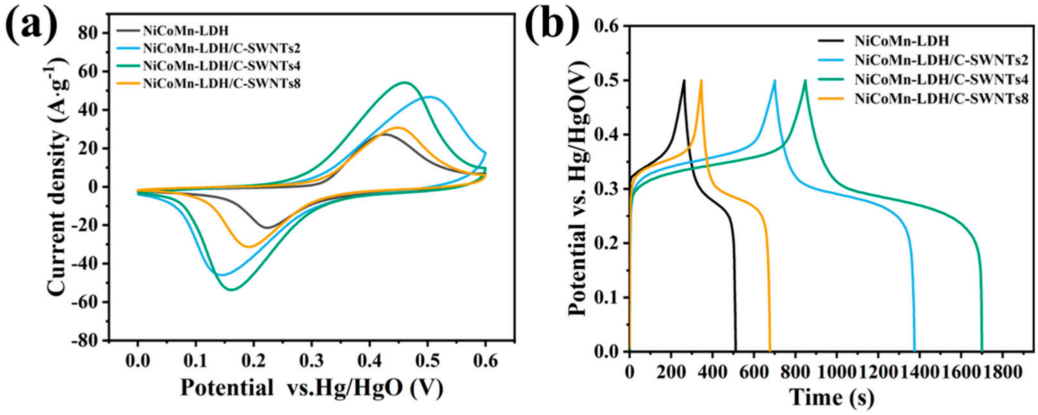

Figure S6. The (a) CV curves at a scan rate of 10 mV s^{-1} and (b) GCD curves at a current density of 1 A g^{-1} for NiCoMn-LDH and NiCoMn-LDH/SWCNTsx (x = 2,4, 8), respectively.

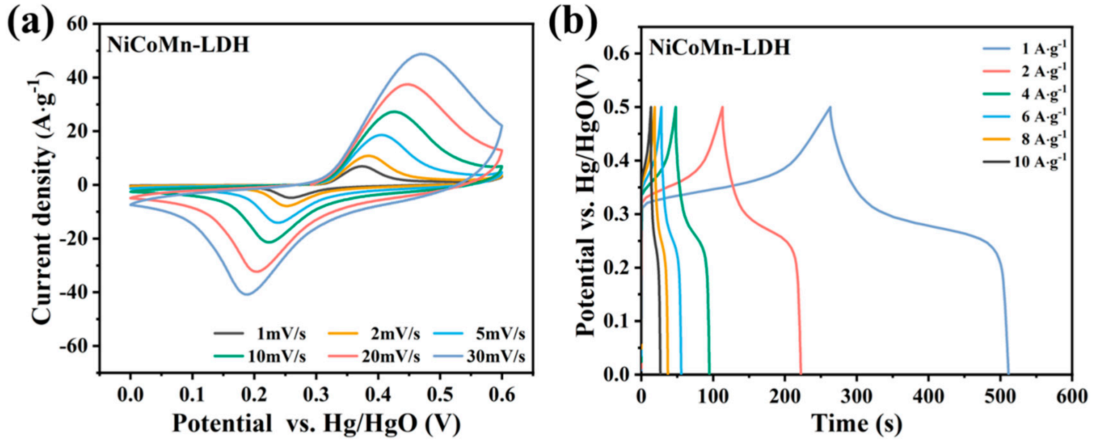

Figure S7. (a) CV curves at 1−30 mV s^{-1} and (b) GCD curves at 1−10 A g^{-1} of NiCoMn-LDH.

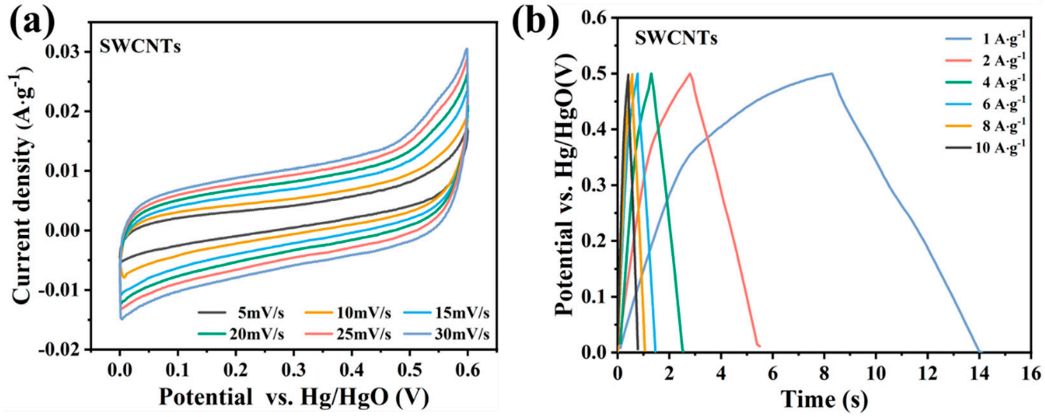

Figure S8. (a) CV curves at 5−30 mV s^{-1} and (b) GCD curves at 1−10 A g^{-1} of SWCNTs

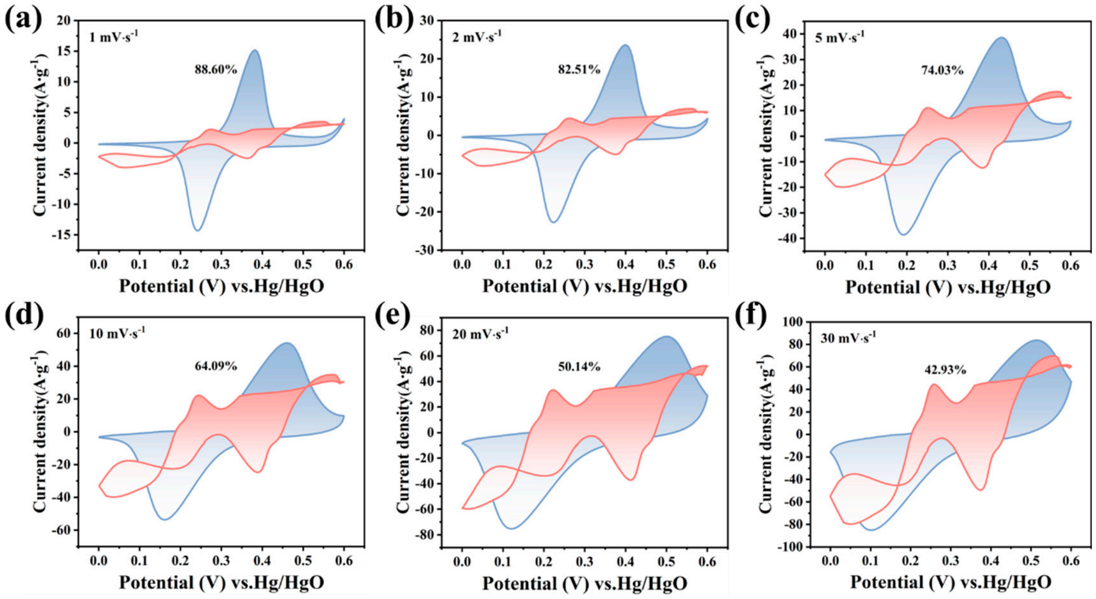

Figure S9. CV curves showing the diffusion contribution for the NiCoMn-LDH/SWCNTs electrode at various scan rates.

Table S1. The BET surface area, average pore size and pore volume of the tested samples

samples	BET Surface Area ($m^2 \cdot g^{-1}$)	Average Pore Size (nm)	pore volume ($cm^3 \cdot g^{-1}$)
ZIF-67	1684.18	1.57	0.026
SWCNTs	368.18	5.84	0.562
ZIF-67/SWCNTs	1738.91	1.62	0.059
NiCoMn-LDH	51.11	17.07	0.15
NiCoMn-LDH/SWCNTs	157.80	14.63	0.43

Table S2. Specific capacitances of NiCoMn-LDH and NiCoMn-LDH/SWCNTsx (x = 2, 4, 8) at various current densities.

Electrode materials	1 A g^{-1}	2 A g^{-1}	4 A g^{-1}	6 A g^{-1}	8 A g^{-1}	10 A g^{-1}
NiCoMn-LDH	496.2	437.2	375.8	330.2	292.6	261.8
NiCoMn-LDH/SWCNTs2	1346.6	1278.5	1194.3	1113.8	1067.2	1007.9
NiCoMn-LDH/SWCNTs4	1704.3	1629.8	1544.7	1480.1	1420.3	1365.6
NiCoMn-LDH/SWCNTs8	661.3	625.42	573.56	527.9	496.7	489.4

Table S3. The capacitive performance of the LDH based electrode materials

Materials	Electrolyte	Specific capacity	Retention rate	Ref
Ni-ZIF-67/Mn-LDH	2M KOH	1420 F g^{-1} at 1 A g^{-1}	78%~1000 cycles	[3]
NiCo-LDHs/RGO	3M KOH	1094 F g^{-1} at 5 A g^{-1}	81%~3000 cycles	[4]
CoS_x@NiCo LDHs	2M KOH	1562 F g^{-1} at 1 A g^{-1}	76.62%~3000 cycles	[5]
NiCo LDH	3M KOH	327 C g^{-1} at 1 A g^{-1}	74.8%~10000 cycles	[5]
NiCr-LDH	2M KOH	296 C g^{-1} at 1 A g^{-1}	80%~5000 cycles	[7]
NiCr-LDHs-POW	2M KOH	736 C g^{-1} at 1 A g^{-1}	86%~5000 cycles	[7]
MnO_2@NiCo-LDH/CoS_2	2M KOH	1547 F g^{-1} at 1 A g^{-1}	82.3%~2000 cycles	[8]
CoNi-LDH@MX-CNF	3M KOH	996 F g^{-1} at 1 A g^{-1}	78.6%~3000 cycles	[9]
NiV-LDH@CNT	6M KOH	1493 F g^{-1} at 1 A g^{-1}	68.8%~1000 cycles	[10]
NiAl-LDH/MXene	3M KOH	1600 g^{-1} at 1 A g^{-1}	78%~3000 cycles	[11]
NiCoMn-LDH	2M KOH	227.8 mAh g^{-1} at 1 A g^{-1}	83.9%~1500 cycles	[12]
Ni_2CoMn_1-LDH	3M KOH	1634.4 F g^{-1} at 1 A g^{-1}	——	[13]
SAC@$Ni_2Co_{0.95}Mn_{0.05}$-LDH	3M KOH	312.61 F g^{-1} at 1 A g^{-1}	83.76%~50000 cycles	[14]
NiCoMn LDH/Ti_3SiC_2	1M KOH	566.6 F g^{-1} at 0.5 A g^{-1}	——	[15]
MXene/CoNiMn-LDH	3M KOH	906 F g^{-1} at 1 A g^{-1}	90.8%~10000 cycles	[16]
IPC/NiCoMn-LDH	6M KOH	2236 F g^{-1} at 1 A g^{-1}	85.9%~5000 cycles	[17]
NiCoMn-LDH/SWCNTs	1M KOH	1704.3 F g^{-1}at 1 A g^{-1}	78.6%~2000 cycles	This work

Table S4 The capacitive performance of the HSC devices reported recently

supercapacitor	Energy density (Wh kg^{-1})	Power density (W kg^{-1})	Retention	Ref
Ni-ZIF-67/Mn-LDH// Ni-ZIF-67/GO-LDH	44	3500	97%~1000 cycles	[3]
CoMn LDH-2//rGO	23.8	300	82.7 %~3010 cycles	[18]
NCA-LDH@NCS@CC//AC@CC	33.13	750	71.4%~10000 cycles	[19]
$Cu_{0.5}Co_{0.5}$-P@$Ni(OH)_2$//AC	40	319.6	73%~6000 cycles	[6]
NiCr-LDHs-POW//rGO	34	1320	86 %~10000 cycles	[7]
NiV-LDHs@ZIF−67//AC	42.3	520.6	120 %~5000 cycles	[20]
CNT/Co_3S_4@NiCo-LDH//AC	58.3	575	90%~3000 cycles	[21]
CNTs@CuCoLDH/BPQD// CNTs@BPQD	62.1	750	79.1%~10000 cycles	[22]
$KNi_{0.8}Mn_{0.2}F_{3-\delta}$//rGO	52.3	800	69%~10000 cycles	[23]
NiCoMn-LDH/SWCNTs//BGA	45.7	700	82.3%~5000 cycles	This work

References

[1] Jiang, X.X.; Zhou, Q.; Lian, Y.F. Efficient photocatalytic degradation of tetracycline on the $MnFe_2O_4$/BGA composite under visible light. *Int. J. Mol. Sci.* **2023**, *24*, 9378.

[2] Zhao, X.; Li, H.; Zhang, M.; Pan, W.; Luo, Z.; Sun, X. Hierarchical nanocages assembled by NiCo-Layered double hydroxide nanosheets for a high-performance hybrid supercapacitor. *ACS Appl. Mater. Interfaces* **2022**, *14* (30), 34781-34792.

[3] Elsonbaty, A.; Harb, M.; Soliman, M.; Ebrahim, S.; Eltahan, A. Metal organic framework/layer double hydroxide/graphene oxide nanocomposite supercapacitor electrode. Appl. Phys. Lett. **2021**, *118* (2). 023901.

[4] Cai, X.; Shen, X.; Ma, L.; Ji, Z.; Xu, C.; Yuan, A. Solvothermal synthesis of NiCo-layered double hydroxide nanosheets decorated on RGO sheets for high performance supercapacitor. *Chem. Eng. J.* **2015**, *268*, 251-259.

[5] Guan, X.; Huang, M.; Yang, L.; Wang, G.; Guan, X. Facial design and synthesis of CoSx/Ni-Co LDH nanocages with rhombic dodecahedral structure for high-performance asymmetric supercapacitors. *Chem. Eng. J.* **2019**, *372*, 151-162.

[6] Zhang, H.; Xiong, T.; Chen, R.; Wang, Y.; Fang, C.; Xu, L.; Liu, C. Liu. High electrochemical performance of $MnCo_2O_{4.5}$ nanoneedles/NiCo LDH nanosheets as advanced electrodes of supercapacitor. *Electrochim. Acta* **2023**, *455*, 142412.

[7] Padalkar, N. S.; Sadavar, S. V.; Shinde, R. B.; Patil, A. S.; Patil, U. M.; Dhawale, D. S.; Bulakhe, R. N.; Kim, H.; Im, H.; Vinu, A.; Lokhande, C. D.; Gunjakar, J. L. Layer-by-layer nanohybrids of Ni-Cr-LDH intercalated with 0D polyoxotungstate for highly efficient hybrid supercapacitor. *J. Colloid Interface Sci.* **2022**, *616*, 548-559.

[8] Wang, X.; Huang, F.; Rong, F.; He, P.; Que, R.; Jiang, S. P. Unique MOF-derived hierarchical MnO_2 nanotubes@NiCo-LDH/CoS_2 nanocage materials as high performance supercapacitors. *J. Mater. Chem. A* **2019**, *7* (19), 12018-12028.

[9] Jiang, H.; Cheng, J.; He, J.; Pu, C.; Huang, X.; Chen, Y.; Lu, X.; Lu, Y.; Zhang, D.; Wang, Z.; Leng, Y.; Chu, P. K.; Luo, Y. Cobalt–nickel layered double hydroxides on

electrospun MXene for superior asymmetric supercapacitor electrodes. *ACS Omega* **2023**, *8* (51), 49017-49026.
[10] Tu, Q.; Zhang, J.; Cai, S.; Zhang, K.; Zhan, H.; Huang, S.; Chen, L.; Sun, X. One-step preparation of NiV-LDH@CNT hierarchical composite for advanced asymmetrical supercapacitor. *Adv. Eng. Mater.* **2022**, *24* (9), 2101174.
[11] Guo, J.; Bian, Z.; Ye, L.; Shang, Y.; Guo, F.; Zhang, Y.; Xu, J. Double layers combined with MXene and in situ grown NiAl-LDH arrays on nickel foam for enhanced asymmetric supercapacitors. *Ionics* **2022**, *28* (6), 2967-2977.
[12] Dai, J.; Li, Z.; Yu, R.; Huang, D. MOFs as template-derived NiCoMn-LDH with a hollow polyhedron structure for high-performance supercapacitors. *J. Alloys Compd.* **2023**, *936*, 168313.
[13] Chen, Y.; Yang, J.; Yu, H.; Zeng, J.; Li, G.; Chang, B.; Wu, C.; Guo, X.; Chen, G.; Zheng, L.; Wang, X. Design and preparation of NiCoMn ternary layered double hydroxides with a hollow dodecahedral structure for high-performance asymmetric supercapacitors. *ACS Appl. Energy Mater.* **2022**, 5 (6), 6772-6782.
[14] Ma, J.; Sun, Q.; Jing, C.; Ling, F.; Tang, X.; Li, Y.; Wang, Y.; Jiang, S.; Yao, K.; Zhou, X. Mesoporous carbon-supported flower-like Mn-doped Ni–Co layered double hydroxides with high cycling capacitance retention for supercapacitors. *CrystEngComm* **2023**, *25* (20), 3066-3078.
[15] Ali, S.; Marwat, M. A.; Khan, M. F.; Adam, K. M.; Din, Z. U.; Karim, M. R. A. Khan, S. Ti_3SiC_2-coupled NiCoMn LDH nanocomposites as positive electrode for high performance supercapacitors. *J. Alloys Compd.* **2023**, *956*, 170229.
[16]Kasirajan, K.; Rajkumar, P.; Kwon, H. G.; Yim, J.H.; Kim, J.; Choi, H. K. Electrostatically self-assembled dual-functional MXene/CoNiMn-LDH composites for biocompatible electrochemical energy storage and non-enzymatic glucose sensor applications. *Appl. Mater. Today* **2024**, *39*, 102263.
[17] Lu, Z.; Zhao, K.; Guo, H.; Duan, L.; Sun, H.; Chen, K.; Liu, J. In situ construction of NiCoMn-LDH derived from zeolitic imidazolate framework on eggshell-like carbon skeleton for high-performance flexible supercapacitors, *Small*

2023, *20*, 2309814.

[18] Prajapati, M.; Kant, C. R.; Jacob, M. V. Binder free cobalt Manganese layered double hydroxide anode conjugated with bioderived rGO cathode for sustainable, high-performance asymmetric supercapacitors. *J. Electroanal. Chem.* **2024**, *961*, 118242.

[19] Li, Y.; Yan, X.; Zhang, W.; Zhou, W.; Zhu, Y.; Zhang, M.; Zhu, W.; Cheng, X. Hierarchical micro-nano structure based NiCoAl-LDH nanosheets reinforced by $NiCo_2S_4$ on carbon cloth for asymmetric supercapacitor. *J. Electroanal. Chem.* **2022**, *905*, 115982.

[20] Wang, G.; Li, Y.; Xu, L.; Jin, Z.; Wang, Y. Facile synthesis of difunctional NiV LDH@ZIF-67 p-n junction: Serve as prominent photocatalyst for hydrogen evolution and supercapacitor electrode as well. *Renewable Energy* **2020**, *162*, 535-549.

[21] Wei, D.; Zhang, Y.; Zhu, X.; Fan, M.; Wang, Y. CNT/Co_3S_4@NiCo LDH ternary nanocomposites as battery-type electrode materials for hybrid supercapacitors. *J. Alloys Compd.* **2020**, *824*, 153937.

[22] Chen, X.; Luo, B.; Ding, J.; Yang, Q.; Xu, D.; Zhou, P.; Ying, Y.; Li, L.; Liu, Y., Kinetics-favorable heterojunctional CNTs@CuCo-LDH/BPQD electrode with boosted charge storage capability for supercapacitor. *Appl. Surf. Sci.* **2023**, *609*, 155287.

[23] Hasan, M.; Sahoo, S.; Kumar, D. R.; Karthik, R.; Dhakal, G.; Lee, J.; Kim, Y. I.; Shim, J. J. Defect engineering of Mn-substituted $KNiF_3$ perovskite fluoride for high-performance asymmetric hybrid supercapacitors. *J. Energy Storage* **2024**, *97*, 112925.
